# IMPACT OF COVID-19 ON THE MENTAL HEALTH OF OLDER SEXUAL MINORITY CANADIANS: A LONGITUDINAL ANALYSIS

**DOI:** 10.1093/geroni/igac059.3037

**Published:** 2022-12-20

**Authors:** Alexandra Grady, Arne Stinchcombe

**Affiliations:** University of Ottawa, Ottawa, Ontario, Canada; University of Ottawa, Ottawa, Ontario, Canada

## Abstract

The COVID-19 pandemic and related restrictions have had negative impacts on mental health. Sexual minorities (i.e., lesbian, gay, and bisexual; LGB people) have been shown to exhibit mental health disparities stemming from minority stress experiences. The purpose of this study was to examine the mental health trajectories of older sexual minority Canadians in comparison to heterosexual Canadians before and during the pandemic. We used data from the Canadian Longitudinal Study on Aging (CLSA), where data were collected from participants at baseline (2011–2015), first follow-up (2015–2018), and two time points during the COVID-19 pandemic (April-December 2020). General Estimating Equations (GEE) were used to model changes in depression symptoms (CESD-10; n=47,728) and loneliness (UCLA 3-item loneliness scale; n=41,698), adjusting for covariates (i.e., age, sex, income, race, education). Results showed that LGB participants reported more symptoms of depression (β=.595, p < .001) and loneliness (β=.313, p < .001) in comparison to heterosexual participants. Mental health outcomes worsened over time for all participants. These findings highlight the impacts of the pandemic on mental health and the need for tailored interventions for older lesbian, gay, and bisexual individuals.

